# Analysis of ocular surface and quality of life in patients with corneal and conjunctival tumors

**DOI:** 10.5935/0004-2749.20230036

**Published:** 2022-03-08

**Authors:** Andressa Miranda Magalhães, Raphaela Paiva Vieira, Thiago José de Moraes Fernandes, Camilla da Silva Rocha, Taciana Higino, Camila V Ventura, Cecília Menelau Cavalcanti

**Affiliations:** 1 Department of Ophthalmology, Fundação Altino Ventura, Recife, PE, Brazil.; 2 Department of Research, Fundação Altino Ventura, Recife, PE, Brazil.; 3 Department of Ophthalmology, Hospital de Olhos de Pernambuco, Recife, PE, Brazil.

**Keywords:** Eye neoplasms, Conjunctival neoplasms, Corneal diseases, Visual acuity, Quality of life, Neoplasias oculares, Neoplasias da túnica conjuntiva, Doenças da córnea, Acuidade visual, Qualidade de vida

## Abstract

**Purpose:**

To evaluate the impact of corneal and conjunctival tumors on the ocular surface and quality of life of patients before and after surgical treatment.

**Methods:**

This prospective study conducted a preoperative and 30- and 90-day postoperative assessment of patients diagnosed with conjunctival and corneal tumors. Demographic data were collected preoperatively. The 12-Item Short-Form Health Survey (SF-12) and Ocular Surface Disease Index (OSDI) questionnaires were applied to assess patients’ quality of life and perception of their vision-related functions. The tear breakup time and Schirmer tests were performed for ocular surface evaluation. The tumor extensions were measured using ImageJ image analysis software.

**Results:**

Twenty-three patients were enrolled. The mean age at examination was 52.8 ± 17.3 years (range: 27-9 years). The most common tumor type was squamous cell carcinoma (61.5%). The patients’ visual acuity improved significantly at 1 month and 3 months (p=0.018 and p=0.036, respectively). No significant differences were found between tear breakup time and Schirmer tests preoperatively and at 3 months postoperatively (p=0.150 and p=0.490, respectively). The SF-12 scores demonstrated significant differences between the preoperative and 30- and 90-day postoperative mental components (p=0.008 and p=0.026, respectively). Tumor extension was 868.7 ± 344.9 pixels (range, 224.6-1481.6 pixels) and were significantly correlated with the preoperative (p=0.011), 30-day postoperative (p=0.017), and 90-day postoperative (p=0.012) SF-12 mental components, as well as the emotional component at the 30^th^ postoperative day (p=0.016).

**Conclusion:**

Patients with corneal and conjunctival tumors improved their ocular symptoms, visual acuity, and the emotional component of their quality of life after surgical excision of the tumor.

## Abstract (pt)

**Objetivo:**

Avaliar o impacto dos tumores cór­neo-conjuntivais na superfície ocular e na qualidade de vida dos pacientes antes e após o tratamento cirúrgico.

**Métodos:**

Este estudo prospectivo conduziu uma avaliação pré-operatória e com 30 e 90 dias de pós-operatório de pacientes com diagnóstico de tumores de córnea e conjuntiva. Os dados demográficos foram coletados no pré-operatório. Os questionários *Health Survey Short-*Form (SF-12) e *Ocular Surface Disease Index* (OSDI) foram aplicados para avaliar a qualidade de vida dos pacientes e a percepção de suas funções relacionadas à visão. Os testes *tear break-up time* (TBUT) e Schirmer foram realizados para avaliação da superfície ocular. A extensão do tumor foi medida usando o programa ImageJ.

**Resultados:**

Vinte e três pacientes foram incluídos. A média de idade foi de 52,8 ± 17,3 anos (27-79 anos). O tipo mais comum de tumor foi o carcinoma de células escamosas (61,5%). A acuidade visual dos pacientes melhorou significativamente em 1 mês e 3 meses (p=0,018 e p=0,036, respectivamente). Não houve diferenças significativas entre os testes *tear break-up time* e Schirmer no pré-operatório e com 3 meses de pós-operatório (p=0,150 e p=0,490, respectivamente). Os escores do SF-12 demonstraram que o componente mental apresentou diferença estatisticamente significante entre o pré-operatório e no 30 e 90 dias de pós-operatório (p=0,008 e p=0,026, respectivamente). A extensão do tumor foi de 868,7 ± 344,9 pixels (intervalo, 224,6-1481,6 pixels) e foram significativamente correlacionados com o componente mental de SF-12 no pré-operatório (p=0,011), 30 (p=0,017) e 90 dias de pós-operatório (p=0,012), e o componente emocional no 30º dia de pós-operatório (p=0,016).

**Conclusão:**

Pacientes com tumores córneo-conjuntivais melhoraram os sintomas oculares, a acuidade visual e o componente emocional da qualidade de vida após a excisão cirúrgica do tumor.

## INTRODUCTION

Conjunctival and corneal tumors (CCT) encompass a wide range of clinical conditions, including melanocytic, vascular, epithelial, lymphoid, inflammatory, and degenerative tumors. The clinical differentiation of these tumors is based on the patient’s medical background, as well as the typical clinical features of the tumor^(1,2)^. Regardless of etiology, CCT can cause symptoms that vary from mild, such as tearing, to more severe, including visual loss and death. Early diagnosis is, therefore, essential to prevent ocular and systemic dissemination, in addition to preserving visual function^(3,4)^.

In recent years, studies have addressed the quality of life of patients with CCT and have highlighted the impact of ocular diseases in the lives of patients by quantitatively investigating the patient’s symptomatology, discomfort, pain, and disability^(5,6)^. These studies have used questionnaires, such as the 12-Item Short-Form Health Survey (SF-12) and Ocular Surface Disease Index (OSDI), to measure symptomatology in a more objective manner at the different stages of disease evolution in the same patient^(7)^.

With respect to ocular oncology, studies investigating the impact of ocular tumors on the quality of life of patients are scarce^(8,9)^. Kopp et al.^(8)^ and Klingenstein et al.^(9)^ described the quality of life of patients with uveal melanoma and, more recently, Mercado et al.^(5)^ compared the quality of life of patients with ocular surface squamous neoplasia (OSSN) that were submitted to surgical excision versus interferon alfa 2b topical treat­ment. However, to date, none of them evaluated the ocular surface in patients with CCT and correlated it with patients’ symptoms and their quality of life before and after surgical excision of the tumor. Thus, this study aimed to analyze the influence of CCT on tear production, ocular symptoms, and quality of life of patients before and after surgery.

## METHODS

This prospective study included patients from the Department of Ocular Oncology of the Altino Ventura Foundation, Recife, Brazil, diagnosed with CCT between October and December 2017. The study was approved by the Ethics Committee of the Altino Ventura Foundation (Protocol number: 2.439.321), and all participants gave written informed consent prior to enrollment.

The inclusion criteria were patients older than 18 years with a clinical diagnosis of CCT who had not yet been treated. Patients who underwent surgery before ocular tumor excision, those who used antiglaucoma eye drops, and individuals who underwent topical chemotherapy, such as mitomycin C, interferon alfa-2b, or 5-fluorouracil, were excluded from the study.

The study included an ophthalmological evaluation and the application of two questionnaires, one to evaluate patients’ ocular symptoms (OSDI) and the other to evaluate patients’ quality of life (SF-12). This assessment was performed prior to surgery and on the 30^th^ and 60^th^ postoperative days (POD). Patients’ sociodemographic characteristics including age and sex, social habits, duration of symptoms, and surgery date were noted for further analysis.

### Ophthalmological evaluation

The ophthalmological examination included best corrected visual acuity (BCVA), slit-lamp examination, Schirmer test I, and tear breakup time (TBUT) test. BCVA was measured using a standardized Snellen chart and converted to log of the minimum angle of resolution (LogMAR) values.

The Schirmer I and TBUT values were categorized for severity based on the Delphi Panel Report^(10)^. The Schirmer I results were noted in millimeters (mm) after 5 minutes and the TBUT evaluation measured the time in seconds.

The results of the Schirmer test were categorized according to the Dry Eye WorkShop I (DEWS I)^(4)^ classification: mild and/or episodic (variable), moderate or chronic episodic (≤10 mm), frequently or constantly severe (≤5 mm), and severe and/or incapacitating and constant (≤2 mm). The TBUT evaluation measured the time in seconds, and the results were classified as mild and/or episodic (variable), moderate or chronic episodic (≤10 seconds), frequently or constantly severe (≤5 seconds), severe and/or incapacitating, and constant (immediate).

### Ocular symptoms questionnaire

Patients were assessed using the OSDI questionnaire that was validated in Brazil in 2012^(5)^. The OSDI enables a rapid and effective quantitative assessment of dry eye. The tool consists of 12 questions and is divided into three parts: visual function (questions 1 to 5), ocular symptoms (questions 6 to 9), and environmental triggers (questions 10 to 12). Each question was classified on a scale from 0 (“none of the time”) to 4 (“all of the time”)^(7,11)^.

### Quality of life assessment

The SF-12 was used to assess patient quality of life. It was validated in Brazil and contains 12 items that evaluate the individual’s self-rated health perception in the 4 weeks prior to assessment^(11)^. Each group of questions is distributed and graduated in scales and evaluates physical function, physical aspect, pain, overall health, vitality, social function, emotional aspect, and mental health. The instrument’s algorithm is used to measure two major components: the Physical Component Summary (PCS) and Mental Component Summary (MCS). The scores in both components range from 0 to 100, with higher scores indicating a better quality of life.

### Tumor extension

The tumor extension was measured by ImageJ image analysis software (NIH, Bethesda, MD, USA) using slit lamp digital photographs. The horizontal and vertical axes of the lesion were manually measured in pixels. A magnification of at least 50% was used to place the measurement lines accurately. All measurements were repeated, and the averages of the major axes were used for statistical analysis.

### Statistical analysis

Statistical analyses were performed using the SPSS software (v.25.0, IBM, Armonk, NY, USA). The sociodemographic data and time from symptoms onset to surgery were presented as mean ± standard deviation (SD). The classification rates for the type of corneal and conjunctival tumors and Schirmer and TBUT tests were described using absolute and relative frequencies. The SF-12 result was calculated using the mean, SD, and minimum and maximum PCS and MCS values. The relationships between the variables were analyzed using the Mann-Whitney, Kruskal-Wallis, and Spearman tests. A p-value <0.05 was considered statistically significant for all the comparisons of this study.

## RESULTS

The study included 26 patients (13 [57.7%] males) with a mean age of 52.8 ± 17.3 years (range, 27-79 years) (Table 1). The mean time between symptoms onset and surgery was 7.1 ± 7.0 months (range, 1-24 months). The most common CCT identified was ocular surface squamous neoplasia (76.9%), represented by squamous cell carcinoma and corneal intraepithelial neoplasia, wich accounted for 16 and 4 cases, respectively (Table 1).

**Table 1:** Sociodemographic data and histopathological diagnoses of patients with corneal conjunctival tumors

Variables	n=26
**Age (years)**	52.8 ± 17.3
**Sex, n**	
Female	11
Male	15
**Corneal conjunctival tumor frequency, n**	
OSSN*: Squamous cell carcinoma (SCC)	16
OSSN*: Corneal intraepithelial neoplasia (CIN)	4
Conjunctival papilloma	4
Conjunctival melanoma	2

* OSSN= ocular surface squamous neoplasia.

By the 90^th^ POD, 3 patients dropped out from the study, resulting in a total of 23 patients who completed the evaluation.

The mean BCVA improved significantly after tumor excision. The initial BCVA was 0.65 ± 0.62 LogMAR, whereas it was 0.48 ± 0.63 LogMAR on the 30^th^ POD and 0.38 ± 0.46 LogMAR on the 90^th^ POD (p=0.018 and p=0.036, respectively).

The average result of tumor extension on its major axis was 868.7 ± 344.9 pixels (range, 224.6-1481.6 pixels). Regarding the tumor size and the SF-12 quality of life questionnaire results, a statistical significance was observed when comparing the tumor size and the preo­perative (p=0.011), 30^th^ POD (p=0.017), or 90^th^ POD (p=0.012) MCS components, as well as between the tumor size and the “Emotional Limitation” component on the 30^th^ POD (p=0.016).

The TBUT and Schirmer I values before and after tumor excision did not differ significantly (Table 2). However, patients’ ocular symptoms decreased significantly when comparing the mean OSDI score before surgery with the mean OSDI score 30 days after surgery (p=0.00) and 90 days after surgery (p=0.02).

**Table 2:** Pre- and postoperative TBUT and Schirmer tests of patients with corneal conjunctival tumors

	Preoperative		30 POD		90 POD		p-value*
	n	%	n	%	n	%	
TBUT							0.150
Normal to mild	14	53.8	13	50.0	4	17.4	
Moderate	7	26.9	7	26.9	8	34.8	
Severe 1	4	15.4	4	15.4	9	39.1	
Severe 2	1	3.8	2	7.7	2	8.7	
Total	26	100	26	100	23	100	
Schirmer I							0.490
Normal to mild	14	53.8	9	34.6	13	56.5	
Moderate	6	23.1	8	30.8	3	13.0	
Severe 1	6	23.1	8	30.8	7	30.4	
Severe 2	0	0	1	3.	0	0	
Total	26	100	26	100.0%	23	100	

* Chi-squared test. POD= postoperative day; TBUT= tear breakup time test.

The preoperative means of the physical and mental components of the SF-12 questionnaire were 40.7 ± 9.4 (range, 19.8-53.1) and 37.3 ± 12.1 (range, 17.0-60.5), respectively. No statistical difference was observed when comparing the preoperative physical component scores with the 30^th^ POD (p=0.292) and 90^th^ POD (p=0.808). However, a significant improvement was observed in the mental component scores on the 30^th^ POD (p=0.008) and 90^th^ POD (p=0.026).

When analyzing the different SF-12 items, the physical function was the only variable that improved significantly in the physical domain on the 30^th^ POD (p=0.010) and 90^th^ POD (p=0.007). The mental and emotional aspects and social function improved significantly on the 30^th^ POD (p=0.014 and 0.048, respectively) and 90^th^ POD (p=0.036 and 0.016, respectively). The overall heath score improved significantly on the 30^th^ POD and the physical limitation on the 90^th^ POD (p=0.033) (Table 3).

**Table 3:** Comparison of quality of life (SF-12) and OSDI scores between preoperative and 30^th^ and 90^th^ postoperative days

SF-12 Instrument	Preoperative	30^th^ POD	p-value**	90^th^ POD	p-value**
Physical component	40.8 ± 9.5 (19.8-3.1)	38.5 ± 6.4 (23.3-47.5)	0.292	40.6 ± 6.5 (28.6-56.4)	0.808
Mental component	37.3 ± 12.2 (17.1-60.6)	43.2 ± 6.7 (30.7-58.3)	**0.008**	43.7 ± 7.5 (25.5-54.2)	**0.026**
Physical Function	41.2 ± 13.2 (22.1-56.5)	46.8 ± 11.4 (22.1-56.5)	**0.010**	46 ± 12.9 (22.1-56.5)	**0.007**
Physical limitation	38.9 ± 11.7 (20.3-57.2)	39.6 ± 12.2 (20.3-57.2)	0.328	44.6 ± 11.4 (24.9-57.2)	**0.033**
Pain	34.8 ± 14.1 (16.7-57.4)	26 ± 11.5 (16.7-57.4)	0.096	29.5 ± 13.5 (16.7-57.4)	0.072
Overall health	44.6 ± 8.4 (18.9-62)	43.7 ± 11.5 (18.9-62)	**0.025**	42.3 ± 15.5 (18.9-62)	0.626
Vitality	36.8 ± 13.5 (27.6-67.9)	40.3 ± 11.8 (27.6-67.9)	0.809	39.4 ± 11.2 (27.6-57.8)	0.583
Emotional limitation	39.5 ± 15.1 (11.3-56.1)	47.3 ± 12.3 (11.3-56.1)	**0.014**	45.9 ± 11.3 (22.5-56.1)	**0.048**
Social function	35.9 ± 19.4 (16.2-56.6)	41.2 ± 14.9 (16.2-56.6)	**0.036**	45.6 ± 13.6 (16.2-56.6)	**0.016**
Mental health	37.2 ± 10.2 (15.8-52.3)	38.8 ± 4.8 (28-46.3)	0.789	40.7 ± 6.6 (28-52.3)	0.503
**OSDI Instrument**	60.6 ± 30.9 (8.3-100)	30.6 ± 22.4 (0-75)	**0.000**	31.1 ± 23.1 (0-67.5)	**0.002**

* Wilcoxon test;

** p-values <0.05 are considered statistically significant and are highlighted in bold.

SF-12= 12-Item Short-Form Health Survey; OSDI= Ocular Surface Disease Index; POD= postoperative day.

## DISCUSSION

Acquired CCT comprise a group of ocular surface tumors that can originate from the squamous epithelium, melanocytes, and/or lymphocytes found in the conjunctival stroma and affect individuals aged 45-67 years^(1-3,12)^.

Although Shields et al.^(14)^ showed that melanocytic tumors were more frequently seen in the United States, OSSN have been reported in countries located near the equator, including both squamous epithelial dysplasia and squamous cell carcinoma, as the more frequent CCT^(1-3,12)^. The higher prevalence of OSSN in the southern hemisphere has been associated with the higher ultraviolet radiation in the region, an important risk factor for developing this tumor^(1,2,12,13)^. The current study included patients from the Northeast of Brazil and identified OSSN as the most prevalent CCT, corroborating these previous reports.

Although OSSN are slow-growing tumors, they can cause extensive local tissue destruction and visual axis obstruction that affect patients’ visual acuity^(1,13,14)^. The mean time between clinical diagnosis and surgical excision of the tumor was 7 months, after which a significant visual improvement was observed at 1 and 3 months after surgical excision.

No significant changes were noted with regards to the objective evaluation of the ocular surface of these patients using both TBUT and Schirmer I tests. A worsening of the corneal lubrication appeared to occur right after surgery and noted at the 30^th^ POD, but a slight improvement was observed on the 90^th^ POD. In addition, patients in the present study with larger tumors and longer disease duration (time between onset of symptoms and excision of the tumor) presented higher Schirmer I values, sometimes exceeding 35 mm, which is the maximum value on the Schirmer strip. This may be explained by the fact that larger tumors are more infiltrative and can cause more irritation to the corneal and conjunctival surfaces^(15)^.

Patients who presented larger lesions showed statistically significant emotional and, mainly, mental aspects before and after the surgical procedure. Larger tumors cause greater aesthetic changes and, as evidenced in the results, greater exacerbation of symptoms, as perceived by the patient. Consequently, a significant positive impact was observed on the patient’s mental and emotional aspects after surgical excision.

Despite no significant improvements in tear production on the TBUT and Schirmer I tests, OSDI data indicate that the patients noticed a considerable improvement in ocular symptoms after tumor excision. To support our finding, Saboo et al.^(6)^ found a significant improvement in patients’ symptoms using the OSDI instrument in patients with graft-*versus*-host disease that presented dry eye. Therefore, our study suggests that the OSDI instrument is more sensitive than the TBUT and Schirmer I tests in detecting early ocular alterations and patients symptoms due to CCT.

The SF-12 questionnaire has been used to assess the quality of life of patients submitted to enucleation due to uveal melanoma, as well as patients with retinal diseases^(9,16)^. In these studies, SF-12 revealed alterations in the physical and mental component scores. Our study is the first to use this instrument in evaluating patients with CCT, although Mercado et al.^(5)^ recently used the SF-36 to assess the quality of life of patients diagnosed with OSSN. In their study, 58% of patients who underwent surgical excision of tumors presented anxiety, whereas 32% exhibited depression. These findings were similar to those on a group of patients treated with topical chemotherapy treatment only that resulted in improved visual function and recreational activities in all patients^(5)^.

In the present study, the mental component was the most affected in patients with CCT and the most significantly improved after surgery, similar to the findings by Mercado et al.^(5)^ This finding may be explained by the psychological pressure that these patients undergo after diagnosis of an ocular tumor^(17)^. Once informed of the complete tumor removal procedure, patients may feel more confident about their recovery and visual prognosis, which reflects in their emotions and, consequently, improves their mental status as observed on the 30^th^ and 90^th^ POD. Therefore, the present study demonstrates that surgical treatment may also play an important role in patients’ daily activities and social interactions. Thus, proper attention must be given to the social and psychological burdens of this disease, as well.

The limitations of the present study included a possible memory bias since we relied on self-reported information regarding patients’ ocular history. Another limitation of the study was that tumor size was not recorded for every patient, and studies have shown that tumor size can interfere with ocular symptoms and signs^(15)^. Moreover, tumor size was measured in pixels and could not be converted to millimeters. However, several ophthalmological studies have used the same unit of measuring ocular structures^(18,19)^. Measuring tumor size in pixels allowed the assessment of tumor extension in order to better understand patients’ quality of life and ocular symptoms.

In summary, our study showed improvements in ocular symptoms, visual acuity, and quality of life, in terms of the mental and, primarily, emotional aspects, after tumor excision in patients with CCT, notably in patients with larger lesions. Moreover, although the TBUT and Schirmer tests did not indicate significant changes between the pre- and postoperative results, the OSDI instrument was able to evaluate for improvements in symptoms in the patients.
